# The proteostatic effects of traffic-derived air pollution on Alzheimer's disease risk

**DOI:** 10.1098/rsob.200146

**Published:** 2020-08-19

**Authors:** Elise A. Kikis

**Affiliations:** Biology Department, the University of the South, 735 University Avenue, Sewanee, TN 37383, USA

**Keywords:** air pollution, Alzheimer's disease, cell stress responses, neuroinflammation, protein folding, proteostasis

## Abstract

Alzheimer's disease (AD) is an age-related neurodegenerative disease and the leading cause of dementia in the elderly. Recent decades have been marked by considerable advances in our understanding of genetic and environmental risk factors and also of the AD mechanism(s) of action. Nonetheless, there is still no cure and the myriad ways AD affects the brain is overwhelmingly complex. Such complexity is manifest in part by the fact that genetic background interacts with the environment, including traffic-derived particulate air pollution, to greatly exacerbate AD risk. Determining the mechanisms by which particulate air pollution acts as an AD risk factor has the potential to reveal yet unknown aspects of AD pathology. This review carefully peels back the layers of complexity to discern whether a unifying disease model, one with proteostasis imbalance at its core, holds up to scrutiny in light of the recent literature. While the data are compelling, it is now time for carefully designed studies to definitively determine whether particulate air pollution acts with ageing, genetic background and other sources of proteotoxic stress to disrupt the delicate proteostasis balance.

## 1. Introduction

Eighty-five per cent of the world's population is exposed to hazardous levels of particulate air pollution, much of it derived from the burning of fossil fuels [1]. This has been shown to have significant deleterious effects on brain health, including increased Alzheimer's disease (AD) risk [2,3]. AD is an age-dependent neurodegenerative disorder for which there is no cure and little by way of treatments. Over the last few decades, substantial progress has been made in the identification of AD risk. We have long known that ageing is by far the strongest risk factor for AD. Now, we know that layered on top of ageing are additional risks brought on by genetics and gene–environment interactions. Of 29 genetic risk factors for AD [4], the strongest, associated with a two- to 10-fold increased risk for the disease compared to the general population, is ApoE4.

Air pollution has been shown to act in an ApoE4-dependent manner to exacerbate or trigger AD symptoms [5]. The mechanisms by which this occurs are under active investigation. An emerging hypothesis in the field suggests that traffic-derived particulate air pollution acts primarily as a source of proteotoxic stress to disrupt the ability of the brain to maintain proteostasis and ensure the health of the proteome [6–8]. The data that support this hypothesis are discussed, including those that describe the detrimental effects of neuroinflammation on proteostasis. To further address this hypothesis by direct experimentation, future studies are proposed, which are expected to reveal whether particulate air pollution interacts with ageing, genetic background and other factors, to disrupt the protein folding environment.

## 2. Proteostasis collapse is a feature of ageing

Proteostasis refers to a healthy cellular state in which protein synthesis, folding, trafficking and clearance are regulated in a manner that minimizes the accumulation of toxic, misfolded, proteins [9]. This regulation is the purview of the proteostasis network representing approximately 2000 unique proteins [10]. Proteotoxic challenges have been shown to disrupt the ability of cells and organisms to maintain proteostasis, resulting in proteome instability and catastrophic protein misfolding. Such challenges include both intrinsic and extrinsic proteotoxicity, with their effects being especially pronounced during ageing [6].

**Figure 1.**
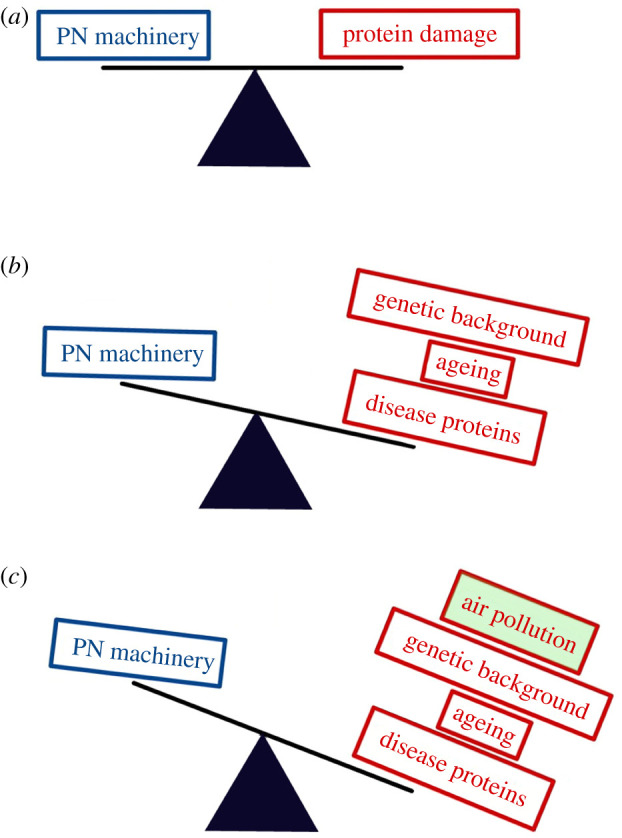
The failure of proteostasis under conditions of high misfolded protein load. When protein damage is minimal, the proteostasis network (PN) machinery is able to clear or refold damaged protein; thereby maintaining a healthy proteome. (*a*) When genetics and ageing increase the misfolded protein load, this can challenge the PN machinery leading to a slightly impaired proteome. (*b*) When internal and external stresses cause the misfolded protein load to exceed the capacity of the PN machinery, proteostasis collapses; thereby disrupting the health of the proteome and leading to cell death and neurodegenerative disease. We propose that air pollution acts a stress on the PN.

An age-dependent decline in the ability to maintain proteostasis is often considered one reason for the late onset of AD and other neurodegenerative diseases [11,12]. Specifically, proteostasis is sufficiently robust early in life to protect against acute bouts of proteotoxic stress (figure 1*a*); however, the capacity of the proteostasis network becomes increasingly limited during ageing when protein damage accumulates (figure 1*b*). This is consistent with findings from *Caenorhabditis elegans* in which both mutant [13] and wild-type [14] proteins suffer catastrophic protein misfolding during ageing. This misfolding induces an age-dependent stress response whereby the FOXO transcription factor, DAF-16 in *C. elegans*, translocates to the nucleus and induces the expression of molecular chaperones and other factors associated with proteotoxic stress [15]. Furthermore, autophagy efficiency [16] and protein translation rates [17] both decline during ageing, resulting in further disruptions to proteostasis.

In addition to ageing, other sources of damaged or misfolded proteins have likewise been shown to contribute to proteostasis imbalance. These include the presence of aggregation-prone disease-associated proteins such as huntingtin and Aβ [18], and polymorphisms in the genetic background that contribute to protein misfolding [19]. Extrinsic factors, including viral infection, also cause significant disruption to proteostasis [20]. Studies have shown that infection with the dengue virus requires Hsp70 molecular chaperones for viral entry and post-entry replication [21]. Likewise, influenza infection was found to disrupt the autophagy arm of the proteostasis network, leading to α-synuclein protein misfolding [22]. Together, the data support the theory that proteostasis collapse during ageing is not caused by a single event. Instead, it stems from a variety of proteotoxic stresses that together increase the misfolded protein load and thereby overwhelm the proteostasis network machinery [23]. Within this framework, it would be reasonable to predict that with the proteostasis network already stressed late in life, any additional damage caused by particulate air pollution would be especially devastating to the ageing proteome. Direct effects of air pollution, such as the oxidation of cellular proteins, may be the final straw for an already stressed proteostasis network, resulting in large-scale protein misfolding, and consequently, the aggravation or onset of AD symptoms (figure 1*c*).

## 3. Proteostasis collapse is a feature of Alzheimer's disease

**Figure 2.**
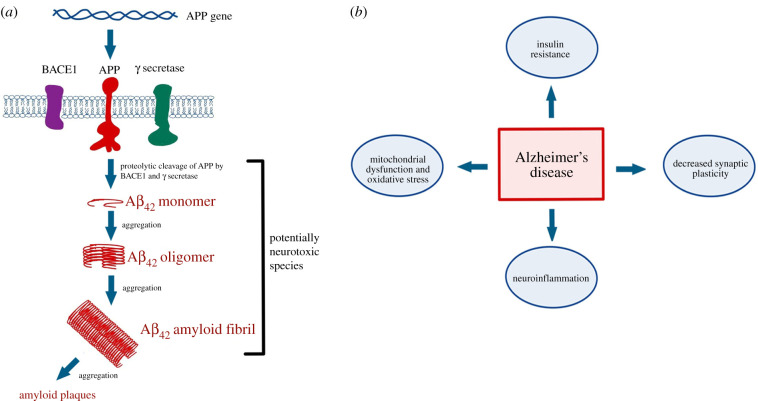
Alzheimer's disease mechanisms of action. (*a*) Studies of familial forms of Alzheimer's disease (AD) revealed mutations in genes/proteins responsible for processing the amyloid precursor protein (APP). Such mutations result in the formation and secretion of amyloidogenic Aβ peptides in certain regions of the brain. The amyloid cascade hypothesis proposes that misfolded and oligomerized or aggregated Aβ is the neurotoxic species that causes AD. (*b*) Studies of sporadic, late- nset, AD have challenged the amyloid cascade hypothesis and revealed additional cellular pathways through which AD may act to exert deleterious effects.

The idea that proteostasis decline upon exposure to particulate air pollution leads to AD onset by disrupting proteostasis assumes that AD is fundamentally a disease of protein misfolding [24]. This assumption is consistent with the amyloid cascade hypothesis, which posits that neurodegeneration experienced by AD patients is caused by the toxic effects of Aβ peptides misfolding and depositing within specific regions of the brain [25]. This was proposed after extensive genetic analyses revealed that the molecular hallmarks of rare monogenic (familial) forms of AD include the misprocessing of the amyloid precursor protein (APP) into amyloidogenic Aβ peptides by membrane-bound proteases and the subsequent formation of amyloid plaques in the brain (figure 2*a*). A second hallmark feature of AD is tau hyper-phosphorylation and the formation of tau neurofibrillary tangles [26]. Tau is a microtubule-associated protein localized to axons and its conversion to a toxic state in the AD brain may be dependent on Aβ [27].

Despite the overwhelming evidence pointing to certain Aβ peptides and hyper-phosphorylated tau being the misfolded and toxic species underlying AD, clinical trials aimed at reducing the Aβ load have been largely unsuccessful. This, and a wealth of additional molecular data on the much more common sporadic forms of AD, has brought the amyloid cascade hypothesis under intense scrutiny. The question laid bare was whether the scientific community has been chasing the wrong target(s). The current thinking is that it may be inaccurate, or at least too simplistic, to explain AD only by the accumulation of toxic and misfolded proteins.

Hungry for new explanations for the cause of neurodegeneration in AD patients, new models were proposed that took into account less-studied aspects of AD pathology. However, instead of entirely upending the amyloid cascade hypothesis, the new models tend to be refinements of the original one—none are entirely independent of the toxic effects of misfolded Aβ. For example, it has been argued that Aβ deposition is triggered by a cycle of mitochondrial damage and ensuing reactive oxygen species (ROS) production [28], a phenomenon that would have the effect of increasing the misfolded protein load. Furthermore, Dourlen *et al*. [29] critically examined the findings of genome-wide association studies (GWAS) and argued that the data largely support the amyloid cascade hypothesis but also point to a role for Aβ and tau in maintaining synaptic plasticity in a manner dependent on the focal adhesion pathway. Ultimately, along with Aβ misfolding, several aspects of neurobiology, especially neuronal plasticity and neuroinflammation, have risen to the forefront of our understanding of AD pathology (figure 2*b*).

## 4. Particulate air pollution may contribute to proteostasis collapse in a manner dependent on neuroinflammation

Chronic neuroinflammation has long been considered a defining feature of AD and other progressive neurodegenerative diseases [30]. However, the mechanisms underlying this phenomenon have recently been re-examined. Kinney and colleagues propose that neuroinflammation is triggered by Aβ-mediated microglial activation early during disease progression, likely during the asymptomatic stages. This seems to be initially protective; however, once inflammation becomes chronic, it ultimately contributes to increased Aβ load and tau hyper-phosphorylation [31]. The neuronal death caused by microglial activation during neuroinflammation has been proposed to be the cause of neurodegeneration and associated dementia in what is referred to as the neuroinflammation hypothesis of AD [32].

The available data suggest that particulate air pollution worsens neuronal dysfunction in AD patients by triggering neuroinflammation. Specifically, mice exposed to airborne particulate matter launched a neuroinflammatory response as evidenced by the activation of key pro-inflammatory transcription factors including NF-κB [33]. At this point, it will be interesting to know whether microglial activation by air pollution occurs via a mechanism that begins with a disruption of the proteostasis balance. One finding in support of a proteostasis-centric mechanism is that particulate air pollution induced neuroinflammation in a manner dependent on the toll-like receptor TLR4 [34], which is activated by binding to damaged proteins. Whether this is damage to Aβ itself, damage to other cellular components (such as lipids) that results in pathological APP processing, or generalized protein damage that causes proteostasis collapse needs to be determined. However, if proteostasis imbalance is at least somewhat involved in TLR4 activation upon exposure to air pollution, such exposure would pose a double threat—that caused by neuroinflammation (a trigger of cell death) and that caused by disrupting the proteostasis balance (catastrophic for the overall health of the proteome). Furthermore, neuroinflammation itself may cause additional stress to an already compromised proteostasis network, leading to a self-sustaining cycle of inflammation and proteostasis decline.

**Figure 3.**
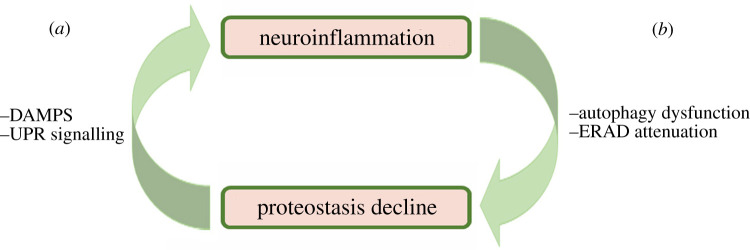
Vicious cycle of proteostasis decline and neuroinflammation. Once either neuroinflammation or proteostasis decline has been triggered, the result is a self-reinforcing cycle that supports an unbalancing of the cellular proteostasis and promotes neuroinflammation. (*a*) Proteostasis decline caused by ageing, damage, or other factors can trigger neuroinflammation via receptors that recognize damage-associated molecular patterns (DAMPS) and also perhaps in a manner dependent on the unfolded protein response (UPR). (*b)* Neuroinflammation has been shown to induce autophagy and attenuate ER-associated degradation (ERAD), both of which would be expected to stress the delicate proteostasis balance.

Consistent with such a cycle, inflammation has been shown to both cause proteostasis imbalance and to itself be caused by such an imbalance (figure 3). Specifically, upon the induction of neuroinflammation via lipopolysaccharide (LPS), autophagy and the unfolded protein response (UPR) were induced, whereas ER-associated protein degradation (ERAD) was attenuated [35]. Likewise, neuroinflammation disrupted the ubiquitin proteasome system (UPS), and bacterial infection of mice induced inflammation in a manner dependent on the UPR [36]. Together, these pathways and cellular processes represent key components of the proteostasis network, including protein degradation pathways (autophagy, UPS, and ERAD) and transcriptional responses to stress (UPR). Completing the cycle, proteostasis imbalance induces inflammation. This depends on the IRE1 branch of the UPR [36] or on the JAK1/STAT3 pathway [37]. Therefore, we should not consider neuroinflammation without at least appreciating its connection to proteostasis.

## 5. Additional evidence of air pollution-mediated proteostasis decline

As described above, we are only beginning to understand how specific branches of the proteostasis network are impacted by exposure to particulate air pollution. However, the data involving nano-sized traffic-derived particulate matter (nPM) or cigarette smoke (CS) are especially telling. For example, a proteomics analysis of lung tissue from smokers and non-smokers revealed an upregulation of the UPR [38], consistent with CS causing oxidative stress in the ER. In fact, the proteostasis network of the lung has been shown to be very sensitive to protein damage [39]. CS also affects the brain, inducing oxidative stress in the rat hippocampus [40]. These findings suggest that oxidative damage to proteins upon exposure to nPM or CS is sufficient to induce canonical transcriptional responses, indicative of an effect on proteostasis. Such challenges to proteostasis in the brain would seem to be a plausible mechanism for increased Aβ deposition under conditions of poor air quality.

Consistent with this hypothesis, Aβ accumulation was increased in the brains of mice exposed to CS [40]. Likewise, traffic-derived nPM acted in an ApoE4-dependent manner in mice, such that mouse models of familial AD that also harbour the ApoE4 allele had more Aβ oligomers in response to nPM than animals lacking ApoE4 [5]. This underscores the fact that gene–environment interactions are capable of inducing a core pathological hallmark of AD. Importantly, the accumulation of Aβ oligomers may be signalling a failure to maintain proteostasis, although this needs to be tested more directly. Another study of mice exposed to nPM revealed a disruption of the protein clearance arms of the proteostasis network [7]. Specifically, when young mice were exposed to nano-sized particulate air pollution, the animals responded with increased levels of proteasome, immunoproteasome and lon protease subunits. Interestingly, the response in older animals was less robust [7], suggesting that a decline in proteostasis during ageing may render older individuals especially susceptible to air pollution-mediated oxidative damage. This is consistent with our initial hypothesis that protein damage during ageing, compounded with environmental exposure, tips the proteostasis balance toward disease (figure 1*c*).

Finally, other branches of the proteostasis network have also been shown to be targets of traffic-derived nPM. Specifically, a recent study using *C. elegans* as a model system revealed that upon acute exposure to nPM during development, oxidative stress-induced transcriptional pathways were activated and shown to be cytoprotective [41]. Additionally, the expression of *hsf-1*, *hsp-4* and *daf-2* were also affected. HSF-1 activates molecular chaperone gene expression upon heat shock and/or protein-folding stress [42]. The *hsp-4* gene encodes one of two *C. elegans* homologues of human BiP, which is an ER molecular chaperone induced via the UPR under conditions of ER stress [43]. Lastly, the *daf-2* gene encodes an insulin-like receptor that controls lifespan and stress responses in *C. elegans* [44,45]. The expression of these genes being dysregulated in response to nPM points to the major protein-folding stress response pathways being significantly impacted in *C. elegans* in response to particulate air pollution.

Although evidence in favour of particulate air pollution causing proteotoxic damage and disrupting branches of the proteostasis network is abundant, it is possible that air pollution also acts by damaging membrane lipids. Specifically, when mice and cultured cells were exposed to nPM, oxidative damage to lipids triggered APP processing and Aβ deposition [46]. However, because oxidative damage to lipids and proteins would happen concurrently, it seems that these two mechanisms may together challenge the proteostasis network to a point where it is overwhelmed and therefore unable to compensate.

## 6. Open questions

We are now at a crucial juncture where it is necessary to systematically dissect the gene–environment interactions that occur in neurons in response to particulate air pollution. As described herein, we now know that exposure to particulate air pollution triggers the accumulation of misfolded and oligomerized Aβ in mice [5], thereby suggesting that the ability to maintain proteostasis is likely compromised. However, we do not know if particulate air pollution causes a generalized decline in overall proteostasis or whether the folding of only specific proteins, such as Aβ, is impaired. One approach to address this important question is to use protein-folding sensors, such as those developed in *C. elegans* [18,47] to monitor changes in protein folding in response to air pollution exposure. Such studies should greatly enhance our understanding of how the environment impacts the health of the proteome and the buffering capacity of the proteostasis network.

We also know that genetic background is a significant contributor to an individual's AD risk, but know very little about how genetic background modulates environmental risk factors. To address this question, we should consider identifying natural genetic variants that modulate the toxic effects of particulate air pollution. Any variation that negatively affects protein folding will likely exacerbate the effects of nPM. This can be tested in laboratory animals using recombinant inbred lines to map susceptibility loci. The laboratories of Morimoto and Gidalevitz have successfully used *C. elegans* to identify natural genetic variation in proteostasis [48,49]. These studies and the aforementioned *C. elegans* models for protein misfolding make *C. elegans* a powerful genetic model to study the effects of particulate air pollution on the ability of cells and organisms to maintain the proteostasis balance. While rapid initial progress can be expected with *C. elegans*, any significant findings should ultimately be tested in rodent models.

In summary, it is our opinion that the existing data provide compelling evidence in favour of proteostasis imbalance being a defining consequence of exposure to particulate air pollution. This imbalance seems to have the effect of enhancing neuroinflammation and protein misfolding in AD. Future studies should directly test this hypothesis and also determine whether air pollution and the presence of metastable protein variants encoded in the genetic background work synergistically to trigger the disruption of proteostasis. Ultimately, answering these questions will enhance our understanding of the AD mechanism of action, thereby bringing us one step closer to designing therapeutic strategies.

## Data accessibility

This article has no additional data.
